# Dietary Bile Acid Supplementation Improves Lipid Metabolism and Selected Egg Quality Traits in Late-Phase Laying Hens Fed a High-Fat Diet and Is Associated with Alterations in Gut Microbiota

**DOI:** 10.3390/ani16162518

**Published:** 2026-08-12

**Authors:** Cai Ma, Mingzhi Liang, Zengguang Wang, Guanghui Teng, Junhai Xue, Jie Ma, Ruili Li, Ming Qin

**Affiliations:** 1 Department of Medical Genetics and Cell Biology, Shandong Medical and Pharmaceutical University, Yantai 264003, China; macai@sdmpu.edu.cn; 2 Institute of Animal Science and Veterinary Medicine, Yantai Academy of Agricultural Sciences, Yantai 265500, China; lmzyt@sina.com (M.L.); wangzengguang126@sina.com (Z.W.); 3 College of Water Resources & Civil Engineering, China Agricultural University, Beijing 100083, China; futong@cau.edu.cn; 4 Yantai Jinlai Food Co., Ltd., Yantai 261413, China; 18553531089@163.com (J.X.); 18561059090@163.com (J.M.)

**Keywords:** bile acid, laying hens, fatty liver hemorrhagic syndrome, lipid metabolism, gut microbiota

## Abstract

High-fat diets may improve energy supply in laying hens, but they can also increase fat deposition in the liver and contribute to fatty liver hemorrhagic syndrome (FLHS), a metabolic disorder that impairs hen health and egg production. Bile acids (BAs), which are natural compounds produced in the liver to support fat digestion and metabolic regulation, may help reduce these negative effects. This study evaluated the effects of dietary BA supplementation on production performance, selected egg quality traits, lipid-related indices, liver histology, and cecal microbiota in late-phase laying hens fed a high-fat diet (HFD). A total of 150 Hy-Line Brown laying hens at 50 weeks of age were allocated either a normal diet (ND), a HFD, or a HFD supplemented with 300 mg/kg BAs (HFD + BAs) for 5 weeks. Compared with the HFD group, BA supplementation affected average daily feed intake (ADFI) and increased the Haugh unit (HU) (*p* < 0.05), whereas laying rate (LR), average egg weight (AEW), feed conversion ratio (FCR), and most other egg quality traits were not significantly changed. BA supplementation was also associated with differences in selected serum and hepatic lipid-related indices. Histological observations indicated differences in hepatic lipid deposition among treatments. Cecal microbiota analysis showed treatment-associated differences in microbial composition, and correlation analysis identified associations between selected taxa and lipid-related indices. These results indicate that, under the present HFD conditions, dietary BAs were associated with changes in selected egg quality traits, hepatic lipid-related indices, and cecal microbial composition. These findings indicate that dietary BAs may improve selected egg quality traits and liver health in late-phase laying hens by regulating lipid metabolism and intestinal microbiota.

## Simple Summary

High-fat feeding may alter lipid metabolism in laying hens and increase hepatic lipid deposition. Bile acids are feed additives that may influence lipid digestion and metabolic processes. In this study, 50-week-old Hy-Line Brown laying hens were fed a normal diet, a high-fat diet, or a high-fat diet supplemented with 300 mg/kg bile acids for 5 weeks. Under the high-fat-diet condition, bile acid supplementation was associated with changes in selected hepatic lipid-related indices, Haugh unit, eggshell color, and cecal microbial composition. However, laying rate, egg weight, and most egg quality traits were not significantly changed. The microbial differences observed were associated with, but do not establish a causal role in, the changes in lipid-related indices. These findings provide preliminary evidence for the potential use of bile acids in diets for late-phase laying hens under high-fat-diet conditions.

## 1. Introduction

Fatty liver hemorrhagic syndrome (FLHS) is a common non-infectious metabolic disorder in laying hens, especially in caged birds with high egg production. It is characterized by excessive lipid deposition in the liver, hepatic enlargement and fragility, and different degrees of hemorrhage, which can reduce laying performance, impair egg quality, increase mortality, and cause economic losses to the poultry industry [1]. In chickens, the liver is the main site of lipid synthesis and is responsible for more than 90% of de novo fatty acid production [2]. During the late laying period, aging and long-term production pressure may reduce hepatic metabolic efficiency, leading to impaired lipid transport and excessive fat accumulation in the liver [3]. Therefore, improving hepatic lipid metabolism is important for maintaining liver health and sustaining egg production in late-phase laying hens.

High-fat diets (HFDs) are often used in poultry production to increase dietary energy density and support production performance [4]. However, excessive dietary fat may overload hepatic and intestinal metabolism when energy intake exceeds the requirements for maintenance and egg formation [5]. Previous studies have shown that high-fat feeding can disturb lipid metabolism, alter gut microbial composition, and promote hepatic steatosis [6,7]. Increasing evidence indicates that the gut–liver axis plays an important role in metabolic liver diseases, as intestinal microorganisms can affect liver lipid metabolism through microbial metabolites, bile acid transformation, immune regulation, and inflammatory responses [8,9]. Thus, nutritional interventions targeting both liver metabolism and gut microbiota may help alleviate HFD-induced fatty liver in laying hens.

Bile acids (BAs) are cholesterol-derived steroid molecules synthesized in the liver and are major components of bile [10]. They are traditionally known as natural emulsifiers that promote the digestion and absorption of dietary lipids and fat-soluble vitamins in the intestine [11]. In addition, BAs act as signaling molecules that regulate lipid metabolism, glucose metabolism, cholesterol homeostasis, energy balance, and inflammatory responses [12]. These effects are mainly mediated through bile acid receptors, including the farnesoid X receptor and G protein-coupled bile acid receptor 1, which are involved in the regulation of fatty acid synthesis, fatty acid oxidation, triglyceride (TG) metabolism, and cholesterol conversion [13]. BAs are also closely linked to intestinal microbiota. After secretion into the intestine, most BAs are reabsorbed and returned to the liver through enterohepatic circulation, while a small proportion reaches the hindgut and is transformed by gut microorganisms [14]. Meanwhile, BAs can selectively shape intestinal microbial communities because of their antimicrobial properties [15]. Conversely, changes in gut microbiota can alter the composition and size of the BA pool, thereby influencing host cholesterol metabolism, TG metabolism, glucose homeostasis, and insulin sensitivity [16]. This bidirectional interaction suggests that BAs may serve as an important bridge between gut microbiota and hepatic lipid metabolism.

Previous studies have shown that dietary BA supplementation can improve lipid digestion, nutrient utilization, growth performance, and lipid metabolism in broilers and fish, particularly under high-fat or high-lipid dietary conditions [17,18]. In laying hens, however, the reported effects of BAs remain inconsistent. Some studies found that BA supplementation improved egg production, feed conversion, serum lipid profiles, and abdominal fat deposition [19,20], whereas others reported limited effects on production performance but reduced hepatic lipid deposition and mortality [21]. These differences may be related to BA source, dosage, diet composition, hen age, and feeding duration. Therefore, the role of exogenous BAs in late-phase laying hens fed a HFD, especially their effects on gut microbiota and hepatic lipid metabolism, requires further clarification.

Although dietary BAs have been reported to influence lipid digestion and metabolic regulation in poultry, evidence in late-phase laying hens exposed to a high-fat diet remains limited. In particular, it remains unclear whether dietary BA supplementation can concurrently modulate hepatic lipid-related indices, selected egg quality traits, and cecal microbial composition under HFD conditions. Therefore, the novelty of the present study lies in the integrated evaluation of these liver-, egg-, and gut-related outcomes in late-phase laying hens challenged with a HFD. The dietary inclusion level of 300 mg/kg BAs was selected based on previous dose–response evidence in late-phase laying hens. Fan et al. reported that supplementation with 300 mg/kg BAs, compared with 200 and 500 mg/kg, significantly reduced hepatic triglyceride content and downregulated hepatic FAS and SCD expression in 76-week-old laying hens, indicating a favorable effect of this dose on hepatic lipid accumulation [22]. In addition, a previous safety study showed that 300 mg/kg porcine BAs did not adversely affect laying performance, serum ALT or AST activities, organ indices, or tissue histopathology in laying hens [20]. Therefore, the present study evaluated the effects of dietary supplementation with 300 mg/kg BAs on production performance, selected egg quality traits, serum and hepatic lipid-related indices, and cecal microbial composition in late-phase laying hens fed a HFD. We hypothesized that BA supplementation would modulate lipid-related indices and cecal microbial composition under HFD conditions.

## 2. Materials and Methods

### 2.1. Animals, Experimental Protocol, and Diets

A total of 150 fifty-week-old Hy-Line Brown laying hens with similar body weights (2.28 ± 0.05 kg) and laying rates (85.10 ± 1.28%) were randomly divided into three groups with five replicates per group and 10 birds per replicate. Prior to the commencement of the trial, all hens underwent a one-week acclimation period with ad libitum access to the basal diet and water. Three groups were treated with the following diets: a normal diet (ND, 3.25% fat), a high-fat diet (HFD, 8.07% fat), and a high-fat diet supplemented with 300 mg/kg bile acids (HFD + BAs, 8.07% fat). Bile acids (BAs), obtained from Shandong Longchang Animal Health Care Co., Ltd. (Dezhou, Shandong, China), contained 7.99% hyocholic acid, 19.65% chenodeoxycholic acid, and 70.88% hyodeoxycholic acid, with a total bile acid content of 98.52%. Dietary fat consisted of soybean oil, and the diet composition is shown in Table 1. Diets were formulated to meet the requirements recommended by the *Hy-Line Brown Management Guide* (Hy-Line, 2018). Each replicate comprised two cages, with five hens per cage. Each wire cage (60 cm × 60 cm × 45 cm) was equipped with individual nipple drinkers and continuous feed troughs. The cages were arranged in a three-tier system, and treatments were distributed across tiers using a balanced randomized block arrangement to minimize potential tier effects. All laying hens were fed three times daily. A stable temperature was maintained between 23 °C and 25 °C, with a relative humidity of approximately 65% throughout the trial period. The hens were allowed ad libitum access to water and feed during the experiment. The henhouse had controlled ventilation and lighting, with 16 h of light followed by 8 h of darkness. The replicate cage was considered the experimental unit for production performance measurements, whereas the individual hen was considered the experimental unit for biochemical parameter, histopathological, and gut microbiota analyses.

### 2.2. Production Performance

The number and weight of eggs were recorded daily at 15:30. Feed consumption was weighed weekly per replicate, along with mortality rates throughout the entire experiment. At the end of the five-week trial, the laying rate (LR), average daily feed intake (ADFI), average egg weight (AEW), and feed conversion ratio (FCR) were subsequently calculated.

LR = Number of eggs laid/number of days to lay eggs × 100%.

ADFI = Total feed intake/Day.

AEW = Total egg weight/number.

FCR = ADFI/AEW.

### 2.3. Sample Collection

At the end of the 5th week, one laying hen from each replicate was randomly selected and weighed for sampling after a 12 h overnight fast. Plasma samples were collected from the wing vein in coagulation-promoting tubes, and then the serum was obtained by centrifuging at 3500 *g* for 10 min at 4 °C and stored at −20 °C. The laying hens were euthanized by severing the jugular vein to facilitate sample collection. Liver tissues were immediately harvested, with a portion fixed in 4% neutral buffered paraformaldehyde for morphological observation and Oil Red O staining, while the remaining liver tissues and cecal content samples were frozen promptly in liquid nitrogen and subsequently stored at −80 °C.

### 2.4. Egg Quality

Ten eggs were randomly collected from each dietary treatment group for egg quality evaluation. Eggs were assessed within 24 h after collection and were maintained at 4 °C before analysis. The egg shape index was calculated by dividing the longitudinal diameter by the transverse diameter with an NFN-1A egg shape index meter (TENOVO FOOD Co., Ltd., Beijing, China). Eggshell thickness was measured non-destructively using a TQ-1A eggshell thickness gauge (TENOVO FOOD Co., Ltd., Beijing, China). Eggshell strength was evaluated with a KQ-1A eggshell strength tester (TENOVO FOOD, Co., Ltd., Beijing, China). Shell color was quantified on the standardized CIE L*a*b* color space using a CR-300 portable colorimeter (Minolta, Co., Ltd., Osaka, Japan): L* denotes relative lightness (0 = black, 100 = white), a* corresponds to the red–green chromaticity axis (positive values indicate redder hue, negative values indicate greener hue), and b* corresponds to the yellow–blue chromaticity axis (positive values indicate yellower hue, negative values indicate bluer hue). Egg albumen height was quantified using an EQ-1A albumen height gauge (TENOVO FOOD Co., Ltd., Beijing, China). Egg weight and Haugh units (HUs) were determined with an EMT-5200 egg multi tester (Robotmation, Co., Ltd., Tokyo, Japan).

### 2.5. Biochemical Parameter Measurement in the Serum and Liver

Enzyme-linked immunosorbent assay (ELISA) was employed to quantify the concentrations of triglycerides (TGs), total cholesterol (TC), low-density lipoprotein cholesterol (LDL-C), and high-density lipoprotein cholesterol (HDL-C) in both serum and liver samples (all assay kits were procured from Shanghai Meilian Biotechnology Co., Ltd., Shanghai, China, and the assays were performed in accordance with the instructions). The following serum analytes were additionally determined via ELISA: insulin (INS), estradiol (E2), progesterone (PROG), follicle-stimulating hormone (FSH), aspartate aminotransferase (AST), and alanine aminotransferase (ALT).

### 2.6. Liver Histology

For histological examination, liver tissues were collected from five hens per treatment, with one hen randomly selected from each replicate. The fixed liver tissues were dehydrated and embedded in an OCT embedding medium. The tissues were cut into 8 m sections using a slicer (LEICA 819, LEICA, Shanghai, China). The sections were then stained with Oil Red O solution in the dark and covered with a lid during the staining process. Next, the sections were sequentially immersed in 60% isopropanol for differentiation. After that, the sections were stained with hematoxylin solution. Finally, the sections were sealed with glycerin gelatin. The stained sections were examined under a microscope (NIKON ECLIPSE E100, Nikon, Tokyo, Japan) and analyzed using an image system (NIKON DS-U3, Nikon, Japan).

### 2.7. 16S rRNA Gene Sequencing

Cecal contents were collected aseptically from one hen per replicate (*n* = 5 per treatment), immediately snap-frozen in liquid nitrogen, and stored at −80 °C until DNA extraction. Total genomic DNA was extracted using a MagPure Soil DNA LQ Kit (Magan, Guangzhou, China) following the manufacturer’s instructions. DNA concentration and integrity were measured with NanoDrop 2000 (Thermo Fisher Scientific, Waltham, MA, USA) and agarose gel electrophoresis. Extracted DNA was stored at −20 °C until further processing. The extracted DNA was used as a template for PCR amplification of bacterial 16S rRNA genes with the barcoded primers and Takara Ex Taq (Takara, Osaka, Japan). For bacterial diversity analysis, the V3–V4 region was amplified via PCR with the following primers: 338F 5′-barcode-ACTCCTACGGGAGGCAGCA-3′ and 806R 5′-GGACTACHVGGGTWTCTAAT-3′. The amplicon quality was visualized using agarose gel electrophoresis. The PCR products were purified with AMPure XP beads (Agencourt, Brea, CA, USA) and amplified for another round of PCR. After purification with the AMPure XP beads again, the final amplicon was quantified using a Qubit dsDNA Assay Kit (Thermo Fisher Scientific, USA). The concentrations were then adjusted for sequencing. Sequencing was performed on an Illumina NovaSeq 6000 with 250 bp paired-end reads (Illumina Inc., San Diego, CA, USA; OE Biotech Company; Shanghai, China).

Library sequencing and data processing were conducted by OE Biotech Co., Ltd. (Shanghai, China). Raw sequencing data were in FASTQ format. Paired-end reads were then preprocessed using Cutadapt software (version 1.9.1) to detect and cut off the adapter. After trimming, paired-end reads were filtered to discard low-quality sequences, denoised, and merged, and chimera reads were detected and cut off using divisive amplicon denoising algorithm 2 (DADA2) with the default parameters of QIIME2 (2020.11). Finally, the software output the representative reads and the ASV abundance table. The representative read of each ASV was selected using the QIIME2 package. All representative reads were annotated and blasted against the Silva database (Version 138) using the q2 feature classifier with default parameters.

### 2.8. Statistical Analysis

The data are represented as the mean ± standard errors of the mean (SEM). One-way analysis of variance (ANOVA) was conducted for statistical testing. The means were compared using Duncan’ s multiple range test, implemented in SAS 9.2 software (SAS Institute Inc., Cary, NC, USA). Statistical significance of differences was expressed by the presence of a–d letters within a column; different letters within a column signify a significant difference between means (*p* < 0.05). Microbial diversity in samples was assessed using alpha diversity metrics. Non-metric multidimensional scaling (NMDS) was performed to visualize similarities and differences in the overall gut microbial community composition among the experimental groups. Statistical differences in community composition were assessed using permutational multivariate analysis of variance (PERMANOVA) based on the Euclidean distance matrix with 9999 permutations. Homogeneity of multivariate dispersion among groups was evaluated using PERMDISP based on the same Euclidean distance matrix. For pairwise PERMANOVA comparisons, *p* values were adjusted using the Benjamini–Hochberg procedure, and adjusted *p* values < 0.05 were considered statistically significant. The linear discriminant analysis effect size (LEfSe) method was employed to compare the taxonomy abundance spectrum, with the top 10 species selected based on relative abundance. The correlation among the abundance of microbiota with serum biochemistry and the hepatic lipids content was conducted by redundancy analysis (RDA) and Spearman correlation analysis, with statistical significance defined as *p* < 0.05.

## 3. Results

### 3.1. Effects of Dietary BAs on Production Performance in Laying Hens Fed with a HFD

**Figure 1:**
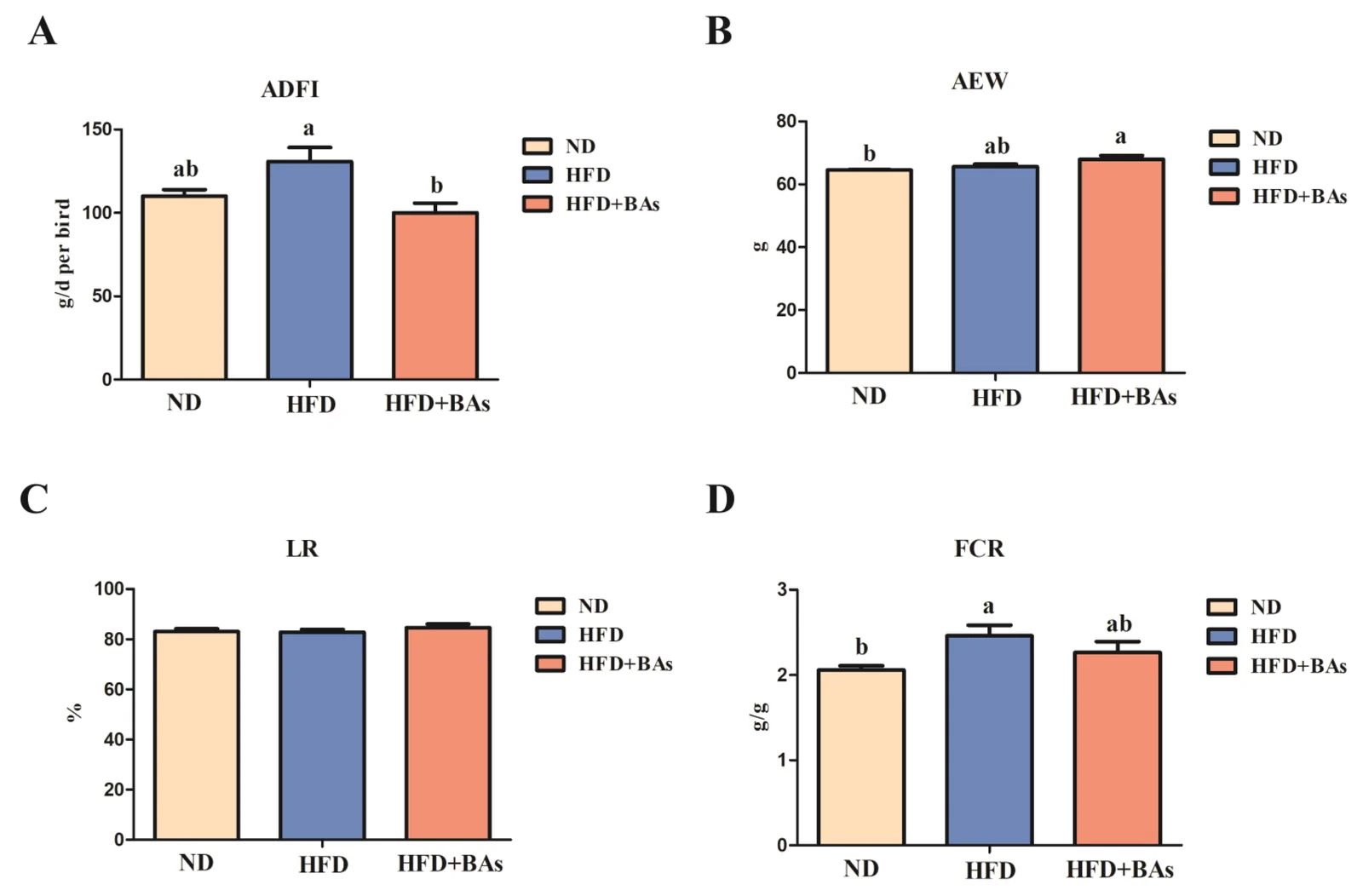
Effects of dietary BAs on production performance in laying hens fed with a HFD. (**A**) Average daily feed intake, (**B**) average egg weight, (**C**) laying rate, and (**D**) feed conversion ratio. Same letters indicate no significant difference (*p* > 0.05), while different lowercase letters indicate significant differences (*p* < 0.05). ^a,b^ Different letters mean significantly different (*p* < 0.05). Abbreviations: ND, a normal diet; HFD, a high-fat diet; HFD + BAs, a high-fat diet supplemented with bile acids; LR, laying rate; ADFI, average daily feed intake; AEW, average egg weight; FCR, feed conversion ratio.

As shown in Figure 1, hens fed the HFD had a higher FCR than those fed the ND (*p* < 0.05), indicating that the HFD reduced feed efficiency. Dietary BA supplementation alleviated this adverse effect. Compared with the HFD group, the HFD + BAs group showed a lower ADFI (*p* < 0.05). However, the LR did not differ significantly among the ND, HFD, and HFD + BAs groups throughout the experimental period (*p* > 0.05). These results indicate that BA supplementation reduced feed intake and improved feed utilization without impairing egg production.

### 3.2. Effects of Dietary BAs on Egg Quality in Laying Hens Fed with a HFD

The effects of dietary BAs on egg quality are presented in Table 2. No significant differences were observed among the three groups in egg shape index, eggshell thickness, eggshell strength, yolk traits, albumen height, or other measured eggshell characteristics (*p* > 0.05). However, the eggshell color b* value was higher in the HFD + BAs group than in the ND and HFD groups (*p* < 0.05). In addition, the Haugh unit was lower in the HFD group than in the ND and HFD + BAs groups (*p* < 0.05).

### 3.3. Effects of Dietary BAs on Serum Biochemical Indices in Laying Hens Fed with a HFD

As shown in Table 3, BA supplementation reduced serum AST and ALT activities compared with the HFD group (*p* < 0.05), indicating an improvement in liver function status. In addition, serum INS and FSH concentrations were increased by BA supplementation (*p* < 0.05). Compared with the ND group, the HFD group showed lower concentrations of LDL-C, PROG, and E2 (*p* < 0.05).

### 3.4. Effects of Dietary BAs on Hepatic Lipid Metabolism and Liver Histology in Laying Hens Fed with a HFD

**Figure 2:**
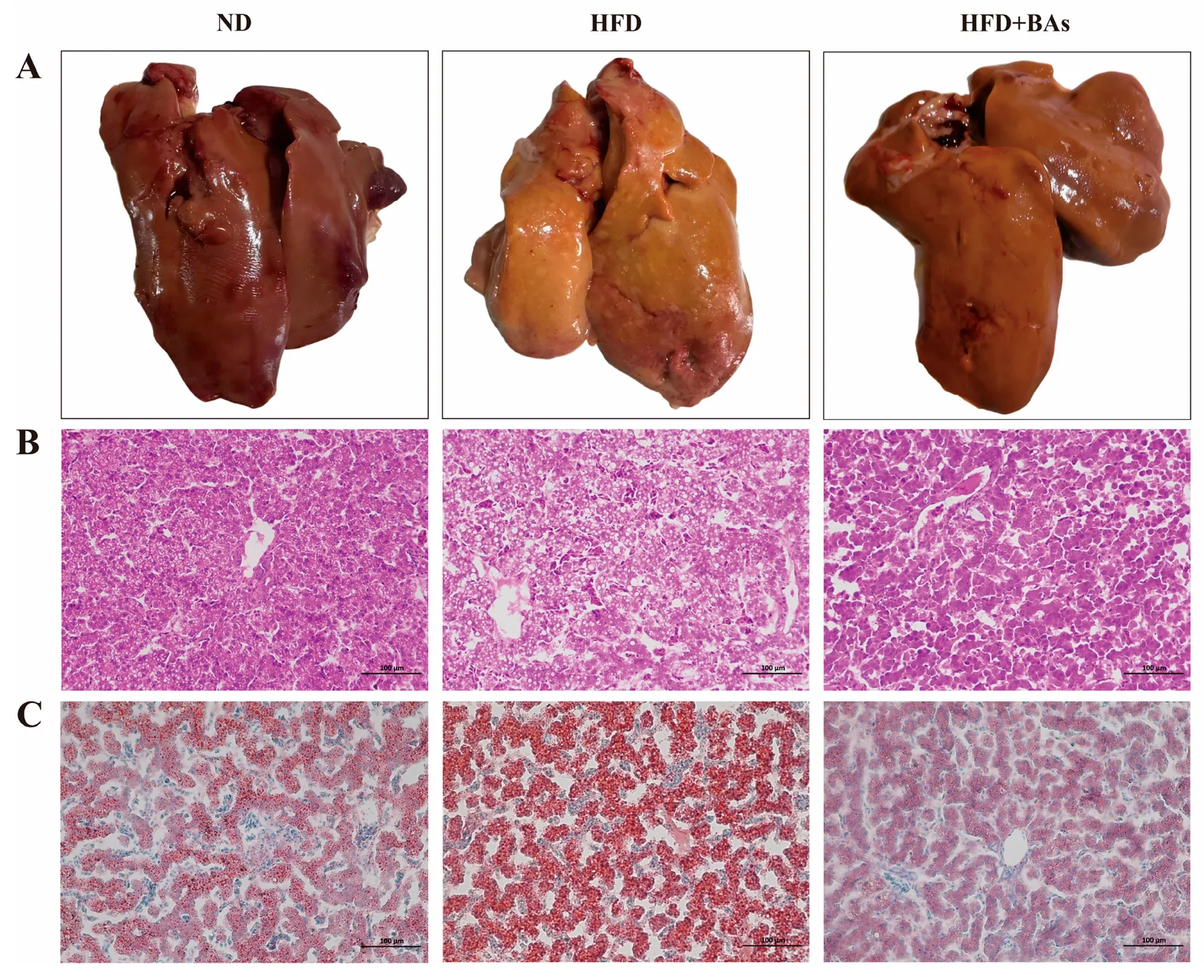
Effect of dietary BAs on liver morphology and histopathology in laying hens fed with a HFD. (**A**) Appearance of the liver after dissection, (**B**) HE staining of liver tissue, and (**C**) Oil Red O staining of liver tissue. Abbreviations: ND, a normal diet; HFD, a high-fat diet; HFD + BAs, a high-fat diet supplemented with bile acids.

The macroscopic liver appearance differed markedly among treatments (Figure 2A). Compared with the ND group, hens fed the HFD showed obvious hepatic abnormalities, including liver swelling, yellowish or grayish-yellow discoloration, hepatomegaly, and increased tissue fragility. Histological examination using hematoxylin and eosin (HE) staining (Figure 2B) and Oil Red O staining (Figure 2C) further showed that a HFD induced severe hepatic steatosis, with extensive lipid droplet accumulation in hepatocytes.

Consistent with the histological observations, hepatic TC, TG, and LDL-C contents were significantly higher in the HFD group than in the other groups (*p* < 0.05) (Table 4). The change in hepatic HDL-C showed an opposite trend to that of TC, TGs, and LDL-C (*p* < 0.05).

### 3.5. Effects of Dietary BAs on Cecal Microbiota Composition in Laying Hens Fed with a HFD

**Figure 3:**
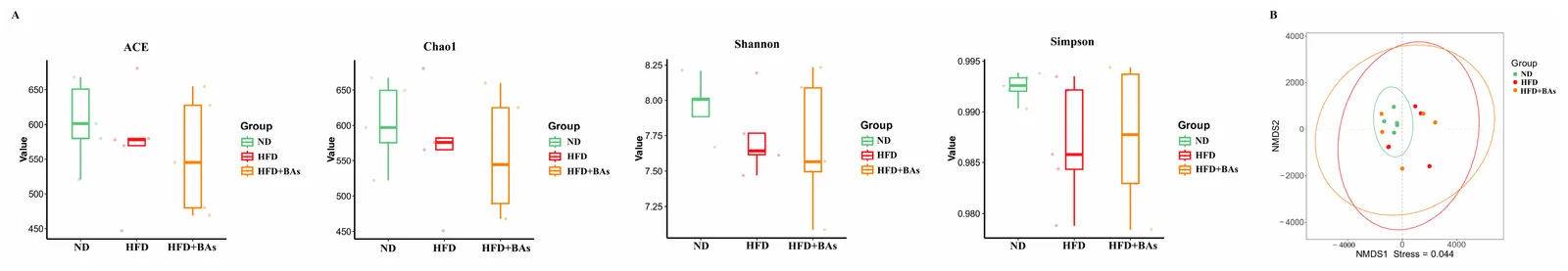
Effects of dietary BAs on the diversity of cecal microbiota in laying hens fed with a HFD. (**A**) Effects of dietary BAs on the ɑ-diversity of cecal microbiota. Boxplots show the differences in ACE, Chao 1, Shannon, and Simpson indices between groups. (**B**) The β-diversity analysis of cecal microbiota among groups. NMDS analyses were performed based on Euclidean distance. Abbreviations: ND, a normal diet; HFD, a high-fat diet; HFD + BAs, a high-fat diet supplemented with bile acids.

The cecal microbiota was analyzed using 16S rRNA sequencing. Alpha diversity indices (Figure 3A) did not differ significantly among the three groups (*p* > 0.05), suggesting that dietary treatments did not markedly affect microbial richness or diversity. However, beta diversity analysis (Figure 3B) showed a clear separation in microbial community structure among groups, indicating that dietary BA supplementation altered the overall composition of the cecal microbiota.

**Figure 4:**
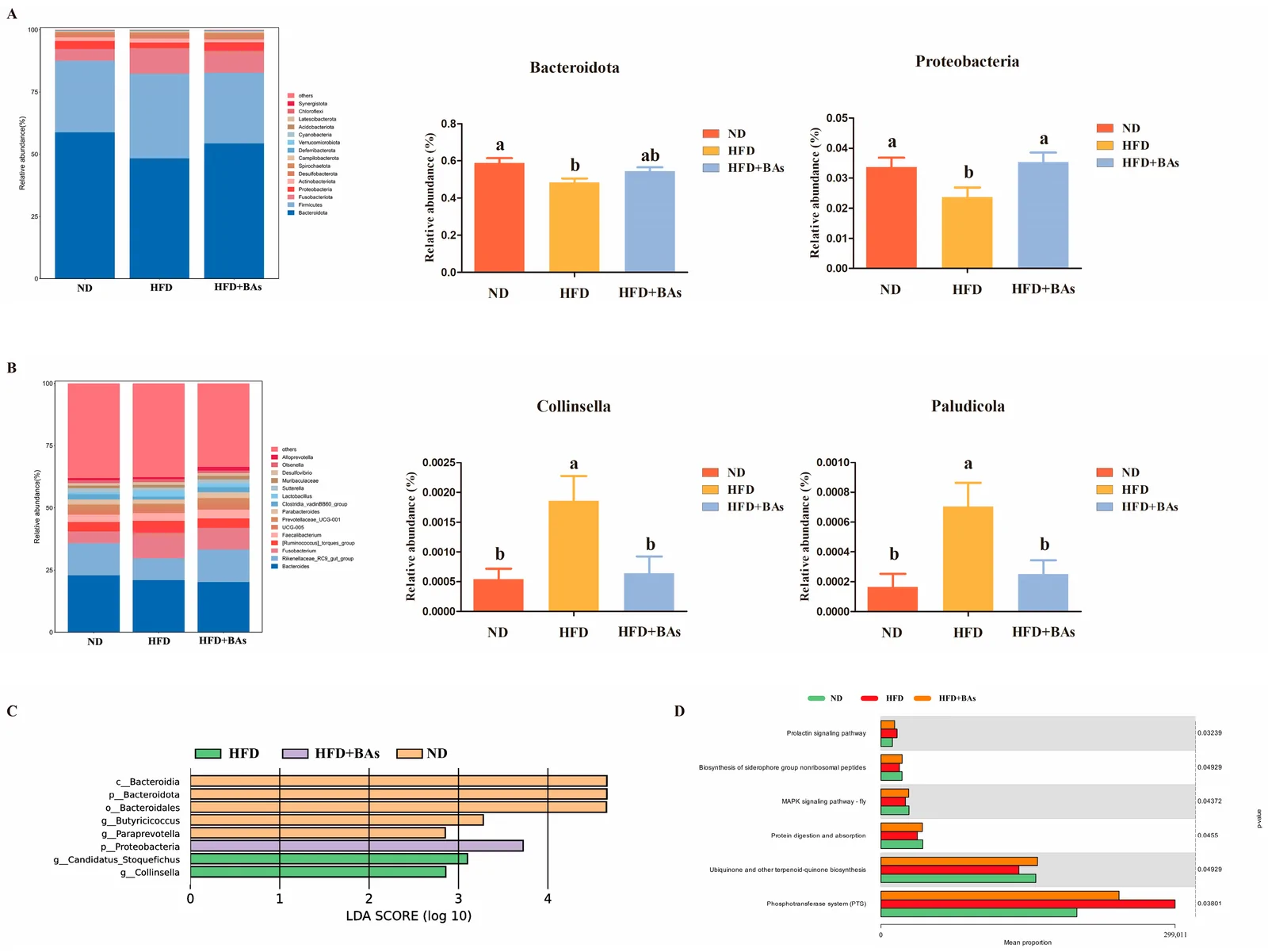
Effects of dietary BAs on changes in cecal microbiota in laying hens fed with a HFD. The classification composition and relative abundance of cecal microbiota in each group at the phylum (**A**,**B**) genus levels,. (**C**) Linear discriminant analysis (LDA) (LDA > 2.0, *p* < 0.05) of cecal microbiomes. (**D**) KEGG pathways of cecal microbiota. ^a,b^ Different letters mean significantly different (*p* < 0.05). Abbreviations: ND, a normal diet; HFD, a high-fat diet; HFD + BAs, a high-fat diet supplemented with bile acids.

At the phylum level (Figure 4A), *Bacteroidota*, *Firmicutes*, *Fusobacteriota*, and *Proteobacteria* were the dominant bacterial phyla in the cecum, together accounting for more than 80% of the total microbial community. Compared with the ND group, the HFD group had a lower relative abundance of *Bacteroidota* (*p* < 0.05). In contrast, BA supplementation significantly increased the abundance of *Proteobacteria* compared with the HFD group (*p* < 0.05).

At the genus level (Figure 4B), the HFD increased the relative abundance of *Collinsella* and *Paludicola*, whereas dietary bile acid supplementation significantly reduced their abundance compared with the HFD group (*p* < 0.05). Linear discriminant analysis effect size (LEfSe) analysis (Figure 4C) further confirmed that *Proteobacteria* were enriched in the HFD + BAs group, whereas Candidatus Stoquefichus and *Collinsella* were enriched in the HFD group.

Functional prediction using PICRUSt2 (Figure 4D) showed that dietary BA supplementation altered the predicted metabolic capacity of the cecal microbiota. Compared with the HFD group, the HFD + BAs group showed increased relative abundance of genes associated with several KEGG level 3 pathways, including the MAPK signaling pathway, protein digestion and absorption, and ubiquinone and other terpenoid-quinone biosyntheses.

### 3.6. Correlations Between Cecal Microbiota and Lipid Metabolism Indices

**Figure 5:**
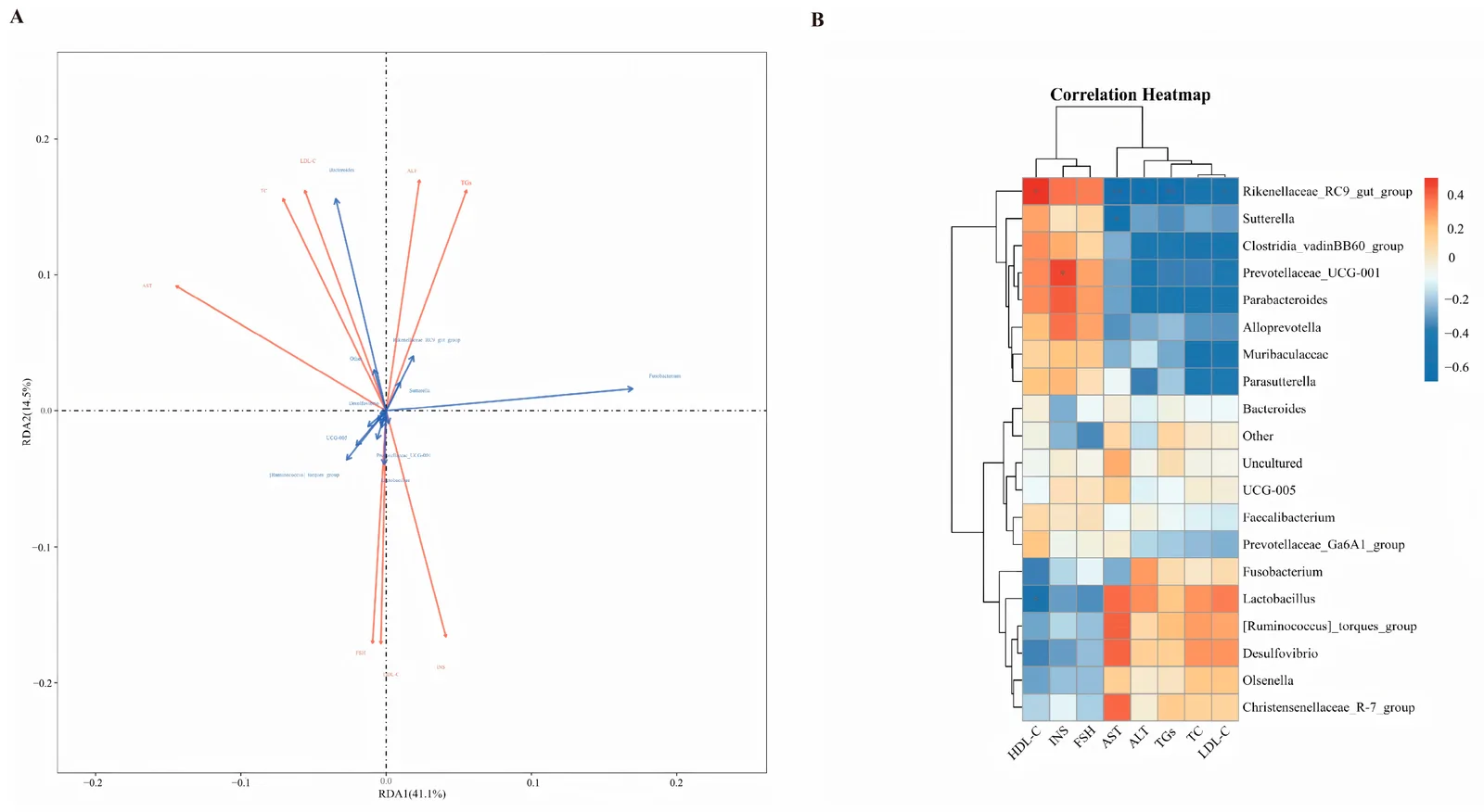
Integrated analysis of lipid metabolism indices and gut microbiota. (**A**) RDA showing the relationships between lipid metabolism indices and the gut microbial community. (**B**) Spearman correlation-based hierarchical clustering heatmap illustrating the associations between different lipid metabolism indices and dominant bacterial genera. Red indicates positive correlations, whereas blue indicates negative correlations. * *p* < 0.05; ** *p* < 0.01. Abbreviations: TC, total cholesterol; TGs, triglycerides; HDL-C, high-density lipoprotein cholesterol; LDL-C, low-density lipoprotein cholesterol; INS, insulin; PROG, progesterone; FSH, follicle-stimulating hormone; ALT, alanine aminotransferase; AST, aspartate aminotransferase.

RDA (Figure 5A) was performed to explore the association between lipid metabolism indices and major bacterial genera. The results showed that lipid-related variables explained part of the variation in cecal microbial community structure. Among these variables, TGs and AST had relatively long vectors, indicating stronger associations with microbial distribution patterns.

Spearman correlation analysis (Figure 5B) further revealed close relationships between specific bacterial genera and serum or hepatic lipid metabolism indicators. The abundance of Rikenellaceae RC9 gut group was negatively correlated with serum ALT, serum AST, hepatic TGs, and hepatic LDL-C, but positively correlated with hepatic HDL-C. Serum AST was also negatively correlated with the abundance of Sutterella. In addition, Prevotellaceae UCG-001 was positively correlated with serum INS concentration.

## 4. Discussion

In the present study, dietary BA supplementation alleviated several adverse effects induced by a HFD in late-phase laying hens. Although LR was not significantly changed among treatments, BA supplementation reduced ADFI and improved FCR compared with the HFD group. This suggests that BAs may improve feed utilization rather than directly stimulating egg production. Fat is an important energy source in poultry diets and can improve feed efficiency, but excessive fat intake may increase the metabolic burden on the liver and promote fatty liver development [23]. Because BAs are amphipathic molecules that emulsify dietary lipids and increase the surface area available for lipase activity, their supplementation may enhance fat digestion and nutrient utilization under high-fat dietary conditions [24]. However, previous studies have reported inconsistent effects of BAs on laying performance. Some studies found improved production performance in late-phase hens [19], whereas others reported no significant effects at certain dosages [21]. These discrepancies may be related to the source, dose, and composition of BAs, as well as hen age, dietary fat level, feeding duration, and the initial metabolic status of the flock.

Egg quality is affected by both diet and age [25,26]. In this study, BA supplementation did not markedly affect most external egg quality traits, such as eggshell strength, eggshell thickness, or egg shape index, but it improved the Haugh unit compared with the HFD group. This indicates that BAs may help maintain albumen quality in hens fed a HFD. Previous studies also reported that BA supplementation improved certain egg quality traits, including yolk color, possibly by promoting the absorption of fat-soluble pigments such as carotenoids [27]. The improvement in the Haugh unit observed in the present study may be associated with better hepatic metabolic status, as the liver plays an essential role in nutrient metabolism and yolk precursor synthesis. Nevertheless, the mechanism by which BAs regulate albumen quality remains unclear and requires further investigation.

The liver is the central organ for fatty acid synthesis and TG metabolism in poultry [28]. Because birds have an underdeveloped intestinal lymphatic system, absorbed lipids are transported mainly through the portal circulation to the liver, making chickens particularly susceptible to hepatic lipid deposition when fed high-fat diets [29,30]. In this study, hens fed the HFD showed obvious hepatic steatosis, including a yellowish liver appearance, increased lipid droplets, and elevated hepatic TC, TG, and LDL-C levels. These findings confirm that the HFD successfully induced hepatic lipid accumulation. In contrast, BA supplementation reduced hepatic TC, TG, and LDL-C contents and improved liver histological structure, as shown by fewer lipid droplets, smaller vacuoles, and reduced steatotic foci. These results are consistent with previous studies showing that BAs reduce hepatic lipid accumulation in laying hens, broilers, and mammalian models [31,32,33]. However, not all studies have reported the same response; for example, dietary supplementation with 100 or 200 mg/kg BAs did not significantly affect hepatic TC and TG contents in one study [34]. Such differences may be explained by variation in BA composition, absolute supplementation level, feeding period, and physiological stage of the hens.

The lower serum TC and TG values observed in the HFD group relative to the ND group were not anticipated and should be interpreted cautiously. Circulating lipid concentrations represent a dynamic balance among intestinal absorption, hepatic synthesis, lipoprotein secretion, tissue uptake, and ovarian yolk precursor formation. In addition, the ND and HFD differed not only in dietary fat but also in calculated energy and crude protein concentrations. Therefore, the serum lipid pattern cannot be interpreted as an isolated effect of dietary fat. This finding may also reflect the specific sampling time point and biological variability and requires confirmation in an isoenergetic, isonitrogenous factorial design.

Serum biochemical indices further supported the protective effect of BAs on lipid metabolism and liver function. In the present study, the HFD + BAs group exhibited lower serum TC and TG concentrations than the ND group, whereas BA supplementation reduced serum ALT and AST activities compared with the HFD group. TC, TGs, LDL-C, and HDL-C are key indicators of lipid metabolism balance [35], while ALT and AST are commonly used markers of liver injury [36]. Therefore, the decrease in serum lipid levels and liver enzyme activities suggests that BAs improved lipid metabolic homeostasis and alleviated HFD-induced liver damage. Mechanistically, BAs may suppress cholesterol synthesis through feedback regulation [37], enhance low-density lipoprotein receptor expression and cholesterol clearance [38], and promote TG degradation and utilization by regulating lipid metabolism enzymes [39]. These effects may explain the reduced serum and hepatic lipid accumulation observed in this study.

The gut–liver axis is increasingly recognized as an important pathway in the development of fatty liver disease [40]. Gut microbiota can regulate host metabolism by extracting energy from nutrients and producing metabolites that affect lipid metabolism, inflammation, and bile acid transformation [41]. In the present study, BA supplementation did not significantly alter alpha diversity, indicating that microbial richness and evenness were relatively stable among treatments. This finding is consistent with studies showing that BAs did not significantly affect alpha diversity in piglets or laying hens [19]. However, beta diversity analysis showed clear differences in microbial community structure, suggesting that BAs mainly reshaped microbiota composition rather than overall diversity.

At the phylum level, BA supplementation increased the relative abundance of *Proteobacteria* compared with the HFD group. Previous evidence suggests that *Proteobacteria* abundance may be negatively associated with hepatic TG accumulation in HFD-fed mice, indicating a potential relationship between this phylum and reduced hepatic lipid deposition [42]. At the genus level, BA supplementation decreased the abundance of *Collinsella* and *Paludicola*, which were enriched in the HFD group. This change may be biologically meaningful because *Collinsella* aerofaciens has been reported to promote systemic inflammation and fat accumulation by metabolizing BAs and producing ammonia, and its abundance is positively correlated with TG and TC levels in patients with non-alcoholic fatty liver disease [43]. *Paludicola* is a relatively new genus within Ruminococcaceae, and its function remains incompletely defined. However, *Ruminococcaceae* has been implicated in fatty acid and bile acid metabolism, and *Paludicola* has been positively associated with lipogenic gene expression and intramuscular fat synthesis in finishing pigs [44,45]. Therefore, the reduction in *Collinsella* and *Paludicola* may be associated with the improvement in hepatic lipid deposition observed after bile acid supplementation.

The predicted microbial functions also suggested that BAs may influence host metabolism through intestinal microbial pathways. PICRUSt2 analysis showed that BA supplementation increased the predicted abundance of genes related to protein digestion and absorption, ubiquinone and other terpenoid-quinone biosyntheses, and other metabolic pathways. Previous studies have suggested that BAs can selectively inhibit or enrich certain bacteria because of their detergent-like properties and antimicrobial effects [46,47]. Therefore, BA supplementation may improve the functional capacity of the gut microbiota and contribute to better nutrient utilization and lipid metabolism. However, functional prediction based on 16S rRNA sequencing has limitations, and the predicted pathways should be interpreted cautiously.

Correlation analysis identified associations between several bacterial taxa and host lipid-related indices. For example, the relative abundance of Rikenellaceae RC9 gut group was negatively correlated with serum ALT and AST activities, hepatic TG content, and hepatic LDL-C content, and positively correlated with hepatic HDL-C content. In addition, Prevotellaceae UCG-001 was positively correlated with serum INS concentration. These observations are consistent with previous reports that certain microbial taxa may be associated with metabolic phenotypes [48]. Nevertheless, because all variables were assessed at a single terminal time point, the present correlations do not establish directionality or causality. In particular, it cannot be determined whether the observed microbial differences contributed to the lipid-related responses to BA supplementation, reflected secondary changes in response to altered host metabolic status, or both.

Overall, dietary BA supplementation was associated with lower serum and hepatic lipid-related indices, reduced hepatic lipid deposition, lower serum ALT and AST activities, and differences in cecal microbial composition in late-phase laying hens fed a HFD. Although several microbial taxa and PICRUSt2-predicted pathways were associated with lipid-related indices, the present findings do not establish that cecal microbiota changes mediated the metabolic responses to BA supplementation. Future studies integrating longitudinal sampling, bile acid and microbial metabolite profiling, metagenomics or metatranscriptomics, and targeted functional validation are needed to clarify the potential contribution of the gut–liver axis.

A limitation of the present study is that gross hepatic hemorrhagic lesions were not systematically scored, and no predefined histopathological scoring system was applied. Consequently, the severity of FLHS-related hepatic pathological changes could not be comprehensively quantified. Nevertheless, the observed changes in hepatic lipid accumulation and lipid-related indices provide evidence of the potential relevance of BA supplementation to hepatic lipid metabolism under HFD conditions. Future studies incorporating standardized gross liver and histopathological scoring systems will help to further characterize its effects on FLHS-related hepatic alterations. Furthermore, the absence of an ND + BAs group precluded assessment of whether the observed responses were specific to the high-fat-diet condition or reflected a general effect of BAs. Future studies should adopt a 2 × 2 factorial design to test the main effects of dietary fat and BA supplementation and their interaction. In addition, only one BA supplementation level (300 mg/kg) was evaluated in the present study. Therefore, dose-dependent responses, the minimum effective dose, and the optimal supplementation level could not be determined. Moreover, measurements were obtained at a single terminal sampling time, which precluded assessment of the temporal dynamics of lipid-related indices, hepatic alterations, and cecal microbial composition during BA supplementation. Future studies should include multiple BA doses and longitudinal sampling at predefined time points to characterize dose–response relationships and the time course of BA-associated metabolic and microbial changes.

## 5. Conclusions

In conclusion, dietary BA supplementation alleviated the adverse effects of a HFD in late-phase laying hens by improving selected egg quality traits, regulating serum and hepatic lipid metabolism, reducing hepatic lipid deposition, and attenuating liver-injury-related enzyme activities. These findings indicate that BAs may serve as an effective nutritional strategy for maintaining liver health and mitigating fatty liver-related metabolic disorders in laying hens under high-fat dietary conditions. Moreover, BA supplementation reshaped the cecal microbiota composition, and the altered microbial profiles were closely associated with changes in hepatic lipid metabolism. This suggests that the protective effects of BAs may be partly mediated through modulation of the gut microbiota–liver axis. Overall, BAs show potential as a practical feed additive to improve metabolic health and welfare in late-phase laying hens, while further studies are needed to clarify the underlying molecular mechanisms and optimize their application in poultry nutrition.

## Figures and Tables

**Table 1:** Ingredient composition and nutrient levels of ND and HFD (air-dry basis, %).

Items, %	ND	HFD
Ingredients		
Corn	63.70	60.70
Soybean meal	21.00	20.00
Soybean oil	1.00	5.00
Shell powder	9.00	9.00
NaCl	0.30	0.30
Premix ^1^	5.00	5.00
Total	100.00	100.00
Nutrient contents, % ^2^		
Crude protein	14.56	14.01
Crude fat	3.25	8.07
Lysine	0.72	0.71
Methionine	0.42	0.42
Methionine + Cysteine (M + C)	0.63	0.60
Metabolizable energy, MJ/kg	11.95	12.67
Calcium	3.51	3.54
Total phosphorous	0.64	0.65
Available phosphorous	0.41	0.42

^1^ The premix contains the following nutrients per kilogram: vitamin A, 108,000 IU; vitamin D3, 3000 IU; vitamin E, 20 IU; vitamin K, 32 mg; vitamin B1, 0.4 mg; vitamin B2, 3.0 mg; vitamin B6, 1.0 mg; vitamin B12, 0.006 mg; biotin, 0.05 mg; pantothenic acid, 12 mg; folic acid, 0.1 mg; nicotinic acid, 7.0 mg; iron, 80 mg; manganese, 100 mg; zinc, 75 mg; iodine, 0.8 mg; and selenium, 0.35 mg. ^2^ Nutrient levels were calculated values. Abbreviations: ND, a normal diet; HFD, a high-fat diet.

**Table 2:** Effects of dietary BAs on egg quality in laying hens fed with a HFD.

Items	ND	HFD	HFD + BAs	*p*-Value
Eggshell color L*	62.10 ± 0.81	62.87 ± 0.50	61.03 ± 0.75	0.177
Eggshell color a*	15.95 ± 0.34	15.51 ± 0.31	16.66 ± 0.50	0.130
Eggshell color b*	26.46 ± 0.62 ^b^	25.69 ± 0.53 ^b^	28.07 ± 0.32 ^a^	0.007
Egg shape index	1.35 ± 0.02	1.35 ± 0.02	1.38 ± 0.01	0.489
Eggshell thickness, mm	0.36 ± 0.01	0.36 ± 0.01	0.35 ± 0.01	0.435
Eggshell properties, %	9.81 ± 0.28	10.33 ± 0.11	10.04 ± 0.19	0.179
Yolk properties, %	28.24 ± 0.97	28.62 ± 0.51	28.57 ± 0.87	0.942
Albumen height, mm	4.76 ± 0.50	3.83 ± 0.23	4.78 ± 0.25	0.056
Haugh unit	63.87 ± 4.73 ^a^	53.35 ± 2.58 ^b^	64.11 ± 2.22 ^a^	0.025
Eggshell strength, N	3.55 ± 0.23	3.40 ± 0.48	3.14 ± 0.19	0.684

Data are presented as the mean ± SEM; *n* = 10 eggs per group. Same letters indicate no significant difference (*p* > 0.05), while different lowercase letters indicate significant differences (*p* < 0.05). ^a,b^ Different letters mean significantly different (*p* < 0.05). Abbreviations: ND, a normal diet; HFD, a high-fat diet; HFD + BAs, a high-fat diet supplemented with bile acids.

**Table 3:** Effects of dietary BAs on serum biochemical indices in laying hens fed with a HFD.

Items	ND	HFD	HFD + BAs	*p*-Value
TC, mmol/L	10.25 ± 0.19 ^a^	8.89 ± 0.29 ^b^	9.49 ± 0.23 ^b^	0.006
TGs, mmol/L	11.03 ± 0.31 ^a^	6.41 ± 0.18 ^c^	7.48 ± 0.25 ^b^	<0.0001
HDL-C, μmol/L	1517.17 ± 67.38 ^b^	1653.83 ± 57.38 ^ab^	1747.34 ± 57.12 ^a^	0.059
LDL-C, mmol/L	8.73 ± 0.20 ^a^	7.24 ± 0.24 ^b^	7.74 ± 0.20 ^b^	0.001
INS, mU/L	38.62 ± 0.82 ^b^	31.80 ± 0.93 ^c^	46.42 ± 1.07 ^a^	<0.0001
PROG, ng/mL	15.32 ± 0.37 ^a^	9.16 ± 0.44 ^b^	8.59 ± 0.42 ^b^	<0.0001
E2, pg/mL	289.80 ± 10.43 ^a^	201.03 ± 19.16 ^b^	170.48 ± 21.05 ^b^	0.001
FSH, mIU/mL	6.90 ± 0.28 ^b^	5.14 ± 0.38 ^c^	9.66 ± 0.26 ^a^	<0.0001
ALT, U/L	39.76 ± 1.49 ^b^	76.02 ± 2.43 ^a^	42.35 ± 3.68 ^b^	<0.0001
AST, U/L	72.39 ± 1.71 ^b^	89.78 ± 3.40 ^a^	55.97 ± 4.42 ^c^	<0.0001

Data are presented as the mean ± SEM; *n* = 5 hens per group. Same letters indicate no significant difference (*p* > 0.05), while different lowercase letters indicate significant differences (*p* < 0.05). ^a,b,c^ Different letters mean significantly different (*p* < 0.05). Abbreviations: ND, a normal diet; HFD, a high-fat diet; HFD + BAs, a high-fat diet supplemented with bile acids; TC, total cholesterol; TGs, triglycerides; HDL-C, high-density lipoprotein cholesterol; LDL-C, low-density lipoprotein cholesterol; INS, insulin; PROG, progesterone; E2, estradiol; FSH, follicle-stimulating hormone; ALT, alanine aminotransferase; AST, aspartate aminotransferase.

**Table 4:** Effects of dietary BAs on hepatic biochemical indices in laying hens fed with a HFD.

Items	ND	HFD	HFD + BAs	*p*-Value
TC, mmol/mg	0.0013 ± 0.0001 ^b^	0.0018 ± 0.0001 ^a^	0.0011 ± 0.0001 ^b^	<0.0001
TGs, mmol/mg	0.0014 ± 0.0001 ^b^	0.0021 ± 0.0001 ^a^	0.0013 ± 0.0000 ^b^	<0.0001
HDL-C, μmol/mg	0.3828 ± 0.0181 ^a^	0.2208 ± 0.0132 ^b^	0.3992 ± 0.0077 ^a^	<0.0001
LDL-C, μmol/mg	0.0009 ± 0.0001 ^b^	0.0015 ± 0.0001 ^a^	0.0007 ± 0.0001 ^b^	<0.0001

Data are presented as the mean ± SEM; *n* = 5 hens per group. Same letters indicate no significant difference (*p* > 0.05), while different lowercase letters indicate significant differences (*p* < 0.05). ^a,b^ Different letters mean significantly different (*p* < 0.05). Abbreviations: ND, a normal diet; HFD, a high-fat diet; HFD + BAs, a high-fat diet supplemented with bile acids; TC, total cholesterol; TGs, triglycerides; HDL-C, high-density lipoprotein cholesterol; LDL-C, low-density lipoprotein cholesterol.

## Data Availability

The datasets generated and/or analyzed during the current study are available from the corresponding author upon reasonable request.
